# Chronic Liver Disease is One of the Leading Causes of Death in Bangladesh: Experience by Death Audit from a Tertiary Hospital

**DOI:** 10.5005/jp-journals-10018-1090

**Published:** 2014-01-22

**Authors:** Mohammed Forhad Abedin, Mohammad Mahfuzul Hoque, Abu Saleh Md Sadequl Islam, Md Forhadul Islam chowdhury, Dulal chandra das, Syeda Anwara Begum, Ayub Al Mamun, Mamun-Al-Mahtab, Salimur Rahman, Anup Kumar Saha

**Affiliations:** 1 Department of Hepatology, Bangabandhu Sheikh Mujib Medical University, Dhaka, Bangladesh; 2 Department of Medicine, Sir Salimullah Medical College and Mitford Hospital, Dhaka, Bangladesh; 3 Department of Hepatology, Bangabandhu Sheikh Mujib Medical University, Dhaka, Bangladesh; 4 Department of Hepatology, Bangabandhu Sheikh Mujib Medical University, Dhaka, Bangladesh; 5 Department of Hepatology, Bangabandhu Sheikh Mujib Medical University, Dhaka, Bangladesh; 6 Department of Medicine, Sir Salimullah Medical College and Mitford Hospital, Dhaka, Bangladesh; 7 Department of Hepatology, Bangabandhu Sheikh Mujib Medical University, Dhaka, Bangladesh; 8 Department of Hepatology, Bangabandhu Sheikh Mujib Medical University, Dhaka, Bangladesh; 9 Department of Hepatology, Bangabandhu Sheikh Mujib Medical University, Dhaka, Bangladesh; 10 Department of Medicine, Sir Salimullah Medical College and Mitford Hospital, Dhaka, Bangladesh

**Keywords:** Chronic liver diseases, Death audit, Bangladesh.

## Abstract

**Background:**

In industrialized countries, the audit has become an integral part of medical care. The experience from developing countries like Bangladesh is still inadequate. This study had been carried out to find out relation among some factors like age, sex, causes, diurenal variation, duration of hospital stay with death and errors in certification process.

**Materials and methods:**

It was a cross-sectional study conducted at the Department of Medicine, Sir Salimullah Medical College (SSMC) and Mitford Hospital from March 2010 to August 2010. Information of consecutive 100 deaths was collected in a predesigned clinical data sheet within half an hour of every occurrence. Necessary data were collected from hospital case records (admission registrar, case files and death certificates) using structured checklist. Patients who were brought dead were excluded from the study.

**Results:**

Among 100 deaths, 48% were males (n = 48) and 52% were females (n = 52). Within this group, 66.7% were males and 33.3% were females. First day (within 24 hours of admission) death accounted for 46% (n = 46) of all death and by the second day 23% (n = 23) of all deaths occurred. The highest underlying cause of death was cerebrovascular diseases (29% of total death), infectious disease contributed 20%, chronic liver disease 13%, malignancy 7%, poisoning 6%, cor pulmonale 5%, while others were 20%.

**Conclusion:**

In this studychronic liver disease was found to be one of the leading causes of death in our hospital and most of them occurred due to hepatic encephalopathy. So, early detection of hepatic encephalopathy and treatment is necessary to reduce hospital mortality.

**How to cite this article:** Abedin MF, Hoque MM, Islam ASMS, Chowdhury MFI, Das DC, Begum SA, Mamun AA, Mahtab MA, Rahman S, Saha AK. Chronic Liver Disease is One of the Leading Causes of Death in Bangladesh: Experience by Death Audit from a Tertiary Hospital. Euroasian J Hepato-Gastroenterol 2014;4(1):14-17.

## INTRODUCTION

Audit in medical practice is defined as the systematic and critical analysis of the quality of medical care, including the procedures used for diagnosis and treatment, the use of resources and the resulting outcome and quality of life for the patient.^1^ The audit involves a criticism of current practice. However, this is well-appreciated that audit is not fault finding but it encourages thoughtful planning which leads to valid information collection and subsequently to informed decision making.^2^ The review of causes of morbidity and mortality in health care facilities is an important exercise with far reaching implications. This form of clinical audit gives a picture of the prevailing disease pattern in the particular community and at the same time looks out for any change in the disease pattern over time.^3^ In ancient years, audit has become an acquired concept in the context of obstetric and other healthcare in both industrialized and developing countries. Death audit is in practice in United Kingdom, South Africa and Malaysia since 1952, 1998 and early 80’s respectively.^4^

Recently, Bangladesh has started maternal, neonatal and child death audit.^5,6^ Death audit in other health sector especially in medicine department is not started yet. Recently, Director General of Health Services, Government of Bangladesh published a circular to maintain death audit in every department of health sector (Public health-2/ESD-01/ information/2008/454). Death audit is important, because, it gives an understanding to what happens and why this happen. This helps to go beyond rates and ratios to determine the inciting factors and to take measures how deaths could have been avoided.^7^

This study was designed to find out relation among some factors like age, sex, causes, diurnal variation, duration of hospital stay with death pattern in adult medicine units, in a tertiary health facility. Major error in death certification as described by World Health Organization (WHO) like mechanism of death listed without an underlying cause, improper sequencing of events and competing cause of death, minor errors like abbreviation, absence of time intervals and mechanism of death followed by underlying legitimate cause of death.^8^

## MATERIALS AND METHODS

This was a cross-sectional study carried out in Department of Medicine, Mitford Hospital, Dhaka, Bangladesh, from March 2010 to August 2010. During this period, a total of 100 consecutive deaths except those who were brought dead included in this study. We developed a network with nurses, internee doctors and midlevel doctors so that one of us could reach the hospital within half an hour of a death. Necessary data were collected from hospital case records, admission registrar and case files. A checklist was designed to record profile of patients, time of admission, diagnosis at the time of admission, time of death and cause of death. All these data were analyzed by SPSS where necessary.

**Fig. 1:**
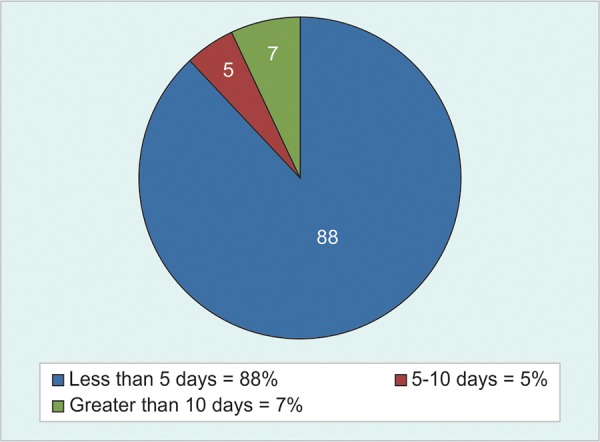
Distribution of death according to duration of hospital stay

**Fig. 2:**
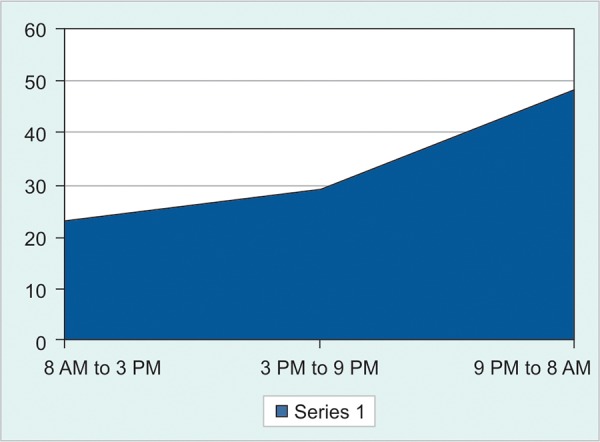
Diurnal variation of death

## RESULT

During the study period, a total of 13,123 (Male-5249, 40%; Female-7874, 60%) patients were admitted in the Department of Medicine, Sir Salimullah Medical College (SSMC) and Mitford Hospital. Among them 100 consecutive deaths in medicine ward were analyzed under death audit. Among 100 deaths, 48% were males (n = 48) and 52% were females (n = 52). The age range was 15 to 85 years. The highest incidence of death occurred in 56 to 65 years group. This group represents 24% of total death. Within this group 66.7% (n = 16) were males and 33.3% (n = 8) were females (Table 1).

On the first day (within 24 hours of admission), death accounted for 46% (n = 46) of all death, by the second day 23% (n = 23) of all death occurred. Before the 5th day, 88% (n = 88) of all death occurred. Only 7% of all death occurred after 10th day (Fig. 1).

During working hour (regular hospital work hours) that means 8 AM to 3 PM, only (n = 23) 23% of death occurred, rest of the deaths (77%, n = 77) occurred after (regular hospital work hours) working hour period. Among the 77% of death that occurred after official hour, 62.3% of death occurred during 9 PM to 8 AM (Fig. 2).

In the present study, during data collection, we observed almost 100% of our existing death certificate, had major errors in a form of mechanism of death listed without an underlying cause, improper sequencing and had 100% minor error in the form of abbreviation, absence of time interval.

According to the audit, the highest underlying cause of death was cerebrovascular disease that was 29% (n = 29) of total deaths. Among the cerebrovascular deaths, as comorbidity hypertension was responsible for 79.3% cases and diabetes mellitus was responsible for 20.7% cases. As an underlying cause of death, infectious disease contributes 20%, chronic liver disease 13%, malignancy 7%, poisoning 6%, cor pulmonale 5%. Table 2 shows the underlying causes of death among the study population.

## DISCUSSION

In our death audit, 88% of all death occurred within 5th day of admission which is consistent with another study conducted in tertiary hospital Kaduna, Nigeria,^9^ where 65% of death occurred within 5th day of admission. It is our limitation that we do not know whether the disease pattern and severity is similar or not in two hospitals. The similarity of result between two studies is due to almost similar socioeconomic background in perspective of healthcare facilities in two countries. First day death contributes a significant portion of a hospital mortality rate even though the hospital can do little to prevent them. Lack of intensive care unit (ICU) facilities and intensive care contributes the most. In absence of ICU facilities, close monitoring of seriously ill patient by better utilization of hospital resources, both human and logistics, can substitute the ICU facilities as it is present in snake bite clinic in Chittagong Medical College Hospital, Chittagong and in malaria ward in Bikaner, India.^10^

**Table 1:** **Table 1:** Age-sex distribution of the study population = 100

*Age (years)*		*Male*		*Female*		*Total*	
15-25		6		8		14	
26-35		3		5		8	
36-45		4		8		12	
46-55		8		9		17	
56-65		16		8		24	
66-75		6		10		16	
76-85		4		3		7	
>85		1		1		2	
Total		48		52		100	

**Table 2:** **Table 2:** Distribution of cases by cause of death and sex

*Cause of death*		*Male*		*Female*		*Total*		*Percentage (%)*	
Cerebrovascular disease		15		14		29		29	
Chronic liver disease		7		6		13		13	
Infectious disease		6		14		20		20	
Chronic kidney disease		2		2		4		4	
Ischemic heart disease		4		0		4		4	
Poisoning		3		3		6		6	
Malignancy		3		4		7		7	
Cor pulmonale		3		2		5		5	
Diabetic keto acidosis		3		1		4		4	
Hypoglycaemia		0		1		1		1	
Undiagnosed		2		3		5		5	
Others		0		2		2		2	

In this study, 62.3% of death occurred during 9 PM to 8 AM which is consistent with another study conducted in Germany from 1987 to 1991.^11^ In our country, availability of healthcare provider and facilities are minimum during this period. To reduce mortality, we can ensure optimum number of healthcare provider and arrange optimum healthcare facilities during this period.

In our study, 100% of death certificate had major error. In a study conducted in Canada major error was found in 32.9% cases.^8^ High incidence of error in death certification was probably due to error from death certificate which was supplied by the government of Bangladesh. Our existing death certificate has only one part, whereas standard death certificate by WHO consist of two parts. First part contains immediate cause and underlying cause sequentially which is absent in our death certificate. There is no part two in our death certificate which indicates the contributory factors of death. There is no space for approximate time interval between onset and death in our existing death certificate. There is also lack of knowledge about the process of death certification among the young doctors. There should be more structured and organized teaching to reduce the error in death certification.

In this study, the underlying cause of most death resulted from cerebrovascular disease (29%). High number of death due to stroke with risk factor like hypertension, diabetes mellitus provides the hint of noncommunicable disease as emerging health problem. In one study conducted in Bangladesh at 2010 found that 66% of death was due to non-communicable disease in adult population.^12^ In the health bulletin of DGHS 2010, the common cause of death has been found to be poisoning in Upazilla hospital.^13^

Although the audit has become an integral part of medical care in industrialized countries, the experience in developing countries yet very rudimentary. However, Government of Bangladesh has taken initiatives to establish perinatal death audit in different hospital since 2004. A decrease in overall mortality rates was recorded after introduction of perinatal mortality audit in LAMB Hospital of Bangladesh (a NGO).^5^ This glorious example should be an eye opening for the professionals, hospital managers and the planners for introducing death audit in a ‘nonblaming’ atmosphere.
